# Synthesis of Molybdenum Sulfide/Tellurium Hetero-Composite by a Simple One-Pot Hydrothermal Technique for High-Performance Supercapacitor Electrode Material

**DOI:** 10.3390/nano11092346

**Published:** 2021-09-09

**Authors:** Hem Prakash Karki, Hyojae Kim, Jinmu Jung, Jonghyun Oh

**Affiliations:** 1 Department of Mechanical Design Engineering, College of Engineering, Jeonbuk National University, Jeonju 54896, Korea; hpkarki666@gmail.com (H.P.K.); hyojaekim@jbnu.ac.kr (H.K.); 2 Department of Nano-Bio Mechanical System Engineering, College of Engineering, Jeonbuk National University, Jeonju 54896, Korea

**Keywords:** nanocomposite, tellurium, hydrothermal, electrochemistry, supercapacitor

## Abstract

It is necessary to investigate effective energy storage devices that can fulfill the requirements of short-term and long-term durable energy outputs. Here, we report a simple one-pot hydrothermal technique through which to fabricate the MoS_2_/Te nanocomposite to be used as an effective electrode material for high-performance supercapacitors. Comprehensive characterization of the as-fabricated nanomaterial was performed using FESEM, HRTEM, XRD, FTIR, XPS, etc., as well as electrochemical characterizations. The electrochemical characterization of the as-fabricated nanocomposite electrode material showed a high specific capacitance of 402.53 F g^−1^ from a galvanostatic charge-discharge (GCD) profile conducted at 1 A g^−1^ current density. The electrode material also showed significant rate performance with high cyclic stability reaching up to 92.30% under 4000 cycles of galvanostatic charge-discharge profile at a current density of 10 A g^−1^. The highly encouraging results obtained using this simple synthetic approach demonstrate that the hetero-structured nanocomposite of MoS_2_/Te electrode material could serve as a promising composite to use in effective supercapacitors or energy storage devices.

## 1. Introduction

Fossil fuels, which are limited natural resources, are being highly exploited due to the urgent need to meet the higher energy demands of society as well as the fast pace of technical advancements in the twenty-first century [1,2,3,4]. The rapid depletion of fossil fuels and the associated environmental pollution must be addressed with the research and development of green and renewable energy technologies for the continuous supply of secure and reliable energy [5,6,7]. Industrial and academic research is currently focusing on the alternative harnessing of energy in an efficient manner to fulfill these energy demands [8]. Among the different types of available devices, batteries and capacitors have attracted substantial attention. Batteries are highly attractive due to their higher energy density, but they possess limited power density compared to that required for the prolonged use of energy. On the other hand, traditional capacitors deliver high power density, but they have low energy storage [9,10,11]. The limitations of both batteries and conventional capacitors, along with the gaps between their performances, can be managed with the intensive exploration of special types of contemporary energy storage tools and techniques, such as electrochemical capacitors, supercapacitors, and ultracapacitors [12]. Supercapacitor devices can be used in various fields—such as in electric vehicles in the automobile industry, military purposes, etc.—due to their fast charge-discharge capacity, high specific capacitance, effective storage capability, good stability, long lifecycle, and significant power density [13,14,15]. These special and unique features of supercapacitors have placed them in the spotlight as alternative energy and power sources to build energy supply systems. The design and development of eco-friendly, high energy density materials for supercapacitor devices would provide an effective and long-term solution to this energy crisis.

Transition metal chalcogenides (TMCs) have attracted substantial attention from researchers due to their typical and interesting physicochemical properties, and they show a wide range of applications, such as energy storage, photovoltaics, and catalysis [16,17]. Among the different TMCs, MoS_2_ nanostructures have attracted significant research attention as supercapacitors due to their high surface area, effective electrochemical activities, excellent supercapacitance, and sufficient electrical conductivities and stability cycles [18,19]. The MoS_2_ layers showed binding of atoms with strong covalent bonding, but the different layers of the MoS_2_ become stacked one over another with the aid of weak Van der Waal bonds. MoS_2_ provides a site of anchor Te NRs with a significant increase in electrochemical performance due to the good stability of the hybrid structure and its excellent electrical conductivity. The crystal structure of the MoS_2_ nanosheets is found to be most stable due to the layered structures of the molybdenum atoms which are sandwiched between two layers of sulfur atoms. The two atomic layers of the molybdenum and sulfur are attracted to each other by the presence of weak Van der Wall force. Similarly, MoS_2_ nanosheets also inhibit the corrosion of other constituent elements present in the composite, thereby ultimately increasing the cyclic stability of the electrode materials. MoS_2_ shows structural similarity to graphene, as it has a 2D sheet-like structure with suitable spacing, and is expected to be useful for exceptional applications in different fields such as in electrocatalysts, super capacitors, sensors, catalysts, absorbent materials, and anti-friction and anti-wear materials [20,21,22].

Tellurium (Te) is one of the interesting p-type semiconductors having very significant features of supercapacitors, mainly due to its very good electrical conductivity and material density. It is widely used in different energy storage systems, such as in Li-Te batteries [23]. Of the metalloids, it shows excellent metallicity, and it also demonstrates a high semiconductor nature in its hexagonal helical-chain conformation in its crystalline structures, along with a small bandgap of 0.35 eV [24]. When used to prepare composite structures with other elements or compounds, Tellurium exhibits additional electrochemical properties [25,26]. Due to its typical properties, a group of researchers synthesized a three-dimensional rGO/Te nanowires composite that showed stable cyclic performance at 0.2 C with a capacity retention of 88% after 200 cycles [27]. Tsai et al. carried out the electrochemical fabrication of Te nanowires, and they found its specific capacitance to be 25 F g^−1^ with a performance retention of 46% [28].

In this work, we applied a simple hydrothermal technique through which to fabricate a nanocomposite of MoS_2_ nanosheets with hexagonal Te NRs to design highly efficient supercapacitance for energy storage devices. To our knowledge, this is a novel design of a MoS_2_/Te composite that has been synthesized for use in supercapacitor devices. The as-fabricated nanocomposite showed a significant supercapacitor value of 402.53 F g^−1^ with a rate of performance of 72.01%. When the electrode material was used for a continuous charge-discharge profile up to 4000 cycles at the current density of 1 A g^−1^, it also showed a good extent of cyclic stability of 92.30%. The high supercapacitance value, good rate capability, and high stability with considerably long lifecycles of the MoS_2_/Te nanocomposite material confirm it to be a prospective electrode material for high-energy storage applications. 

## 2. Materials and Methods

### 2.1. Materials and Reagents

All the chemicals and reagents used in this work were of analytical grade. Sodium tellurite (Na_2_TeO_3_), polyvinylpyrrolidone (PVP, Mw 40 kDa), N-methyl-2-pyrrolidine (NMP), ammonium hydroxide (NH_4_OH), hydrazine monohydrate (NH_2_NH_2_·H_2_O), potassium hydroxide (KOH), ammonium molybdate (NH_4_)_2_MoO_4_, and thiourea (NH_2_CSNH_2_) were all purchased from Sigma Aldrich (Sigma-Aldrich, St. Louis, MO, USA). The chemicals were used as obtained without further purification. During all the experimental procedures, DI-water obtained from minipure RO was used.

### 2.2. Preparation of MoS_2_/Te Composite

A simple one-pot hydrothermal process was used to synthesize the MoS_2_/Te nanocomposite. In detail, 0.784 g of ammonium molybdate was put in a 50 mL glass bottle, to which 0.609 g thiourea was added, followed by the addition of 40 mL DI-water. The mixture solution was left for sonication for 30 min. Next, 92.2 mg sodium telluride and 1.00 g of polyvinylpyrrolidone (PVP, MW 40 kDa) were added into another glass vial, into which 3.33 mL of ammonium hydroxide and 1.67 mL hydrazine monohydrate were poured, and the vial was then shaken for some time. At this point, the solution mixtures of both vials were poured into a 100 mL Teflon lined vessel, then autoclaved at 195 °C for 24 h. The as-synthesized composite nanoparticles were successively washed with DI-water and ethanol to remove any unwanted chemicals, and they were separated by a centrifuge for 20 min at 4000 rpm. The washed nanocomposite was dried in a vacuum oven at 60 °C for 12 h. Similarly, pure MoS_2_ and pure Te were also prepared under similar conditions using only their respective chemical precursors. 

### 2.3. Characterization

The surface morphology of the nanocomposite was analyzed through field emission scanning microscopy (FESEM, S7400, Hitachi, Chiyoda, Tokyo, Japan) operated at an acceleration voltage of 5 kV as well as transmission electron microscopy (TEM-2010, Orius SC10002, JEOL Ltd., Akishima, Tokyo, Japan). The crystalline nature and phase of nanocomposite were investigated by X-ray diffraction (XRD) with Cu Kα, λ Å = 1.540 (Rigaku, Tokyo, Japan). Similarly, Fourier transform infrared (FTIR) spectra were taken by an Bomen MB100 spectrometer (ABB, Zürich, Switzerland), and X-ray photoelectron spectra(XPS) were collected with an A1–Kα irradiation source (Kratos AXIS-NOVA, Shimadzu, Kyoto, Japan). Nitrogen adsorption–desorption was conducted to analyze the specific surface area of the sample through Brunauer-Emmett-Teller (BET) adsorption/desorption isotherm measurements at 77 K (ASAP 2420, Micromeritics, Norcross, GA, USA).

### 2.4. Electrochemical Characterization

The electrochemical properties of the composite nanoparticles were examined on a VersaSTATE-3 electrochemical workstation (AMETEK Inc., Berwyn, PA, USA) at room temperature. For the electrochemical experiments, a three-electrode configuration was maintained with 2 M KOH solution as the electrolyte, Ag/AgCl as the reference electrode, Pt-wire as the counter electrode, and one of the as-prepared composite particles loaded on Ni-foam as the working electrode. Prior to the formation of the working electrode, the Ni-foam was thoroughly washed with 3 M HCl, acetone, DI-water, and absolute ethanol to remove any impurities or oxide films present on its surface. The supplementary information (SI) describes the detailed process for the formation of the working electrode. The cyclic voltammetry (CV) measurements were carried out in the potential range from 0–0.6 V vs. Ag/AgCl as the reference electrode with different scan rates, and galvanostatic charge–discharge (GCD) experiments were conducted within the potential range 0.00–0.45 V vs. Ag/AgCl as the electrode at different current densities ranging from 1–10 A g^−1^. Electrochemical impedance spectroscopy (EIS) was performed at open circuit potentials ranging from 10^−2^ to 10^5^ Hz with an alternating current amplitude of 5 mV, and the results are shown in the form of a Nyquist plot.

The specific capacitance of the as-fabricated electrode material was calculated from the CV profile of the electrode materials using Equation (1) [29,30]:

(1) $$C=\frac{\int Id\Delta V}{ʋ\Delta Va}$$

where C is the specific electrochemical capacitance (F g^−1^), *I* is the applied current in amperes (A), Δ*V* is the potential range of the CV profile, ʋ is the scan rate (V s^−1^), and *a* is the active area of the electrode material (cm^2^).

Similarly, the specific capacitance (*C*) of the electrode material was calculated from GCD profiles based on Equation (2) [31,32]:

(2) $$C=\frac{I\Delta t}{m\Delta V}$$

where *I* is the discharge current in amperes (A), Δ*t* is the discharge time in seconds (s), *m* is the active mass of the electrode material deposited on Ni-foam in grams (g), and Δ*V* is the potential range of the GCD profile.

## 3. Results and Discussion

### 3.1. Morphological Analysis

**Figure 1:**
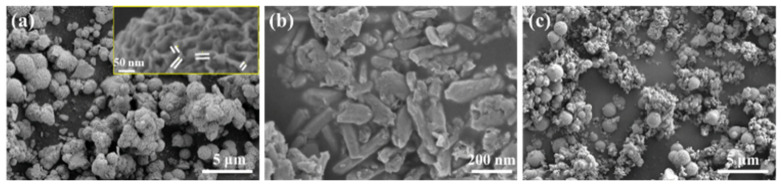
FESEM image of: (**a**) Pure MoS_2_, (**b**) pure Te nanorods, and (**c**) MoS_2_/Te nanocomposite.

Nanocomposite materials were successfully prepared by a simple one-pot hydrothermal technique. During synthesis, the MoS_2_ was produced as nanospheres with an average diameter of ~1 µm, as illustrated in Figure 1. The standard ImageJ software was used to analyze the FESEM images of the samples. Figure 1a shows the FESEM image, which depicts the MoS_2_ nanospheres that have been produced through the hierarchical agglomeration of MoS_2_ nanosheets [33]. The spherical structures obtained through nanosheet collection showed a sheet diameter in the range from (8–16) nm, as shown in the inset of Figure 1a. Similarly, the pure Te nanorods (Te NRs) were produced with the length of (250–540) nm and the diameter in the range from (50–110) nm with clear hexagonal sharp edges, as shown in Figure 1b. Similarly, Figure 1c indicates the successful formation of the hetero-structured nanocomposites of MoS_2_ and Tellurium nanorods (Te NRs). The MoS_2_/Te composite was successfully synthesized through a simple one-pot hydrothermal method. Figure S1 of the SI shows the elemental mapping of the composite that was carried out, which demonstrated the successful formation of MoS_2_/Te hetero-structured nanocomposite from its constituent elements by applying the simple one-pot hydrothermal method.

**Figure 2:**
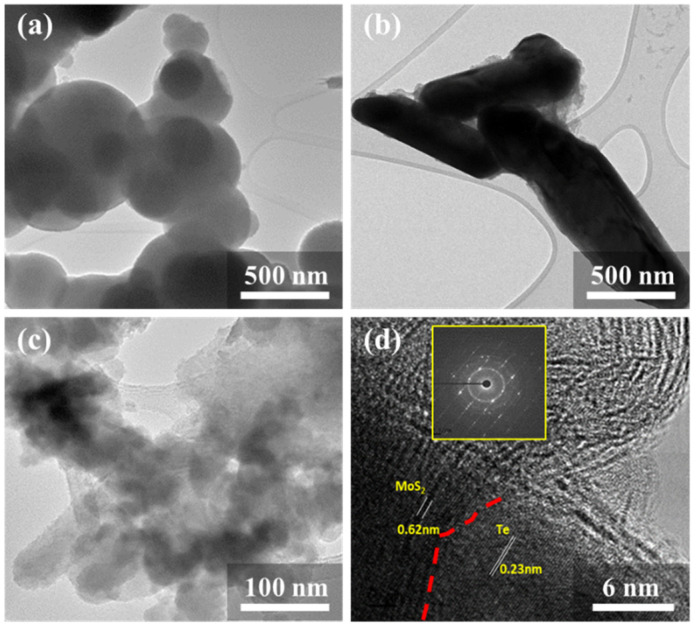
TEM images of: (**a**) Pure MoS_2_, (**b**) pure Te nanorods, and (**c**,**d**) MoS_2_/Te.

Figure 2 shows the high-resolution transmission electron microscopy (HRTEM) that was also used for the morphological analysis of the as-fabricated hetero-structured nanocomposite. The Standard Digital Micrograph software was used to measure the sizes and diameters of the nanocomposites from the HRTEM images. Here, Figure 2a clearly shows the formation of MoS_2_ nanospheres with the diameter in the range from (340 to 680) nm. Similarly, the pure Te NRs were synthesized as a hexagonal crystal structure, which showed an average diameter in the range from (208 to 400) nm with length in the range from (560 to 1220) nm, as shown in Figure 2b. The formation of MoS_2_ nanospheres and Te NRs was also verified by the FESEM images previously presented in Figure 1.

Figure 2c shows that the final nanocomposite of MoS_2_/Te experienced somewhat of a decrease in the size of its components, which was attributed to the presence of a higher molar concentration of the precursor compounds. Crowding of the precursor elements shows the change in the surrounding chemical environment, which is responsible for the decreased dimension of the synthesizing components [34]. Figure 2d shows the HRTEM image of the composite with clear d-spacings of 0.62 nm for MoS_2_ and 0.23 nm for the Te NRs [25,35], and the inset shows the Fast Fourier transform (FFT) image.

**Figure 3:**
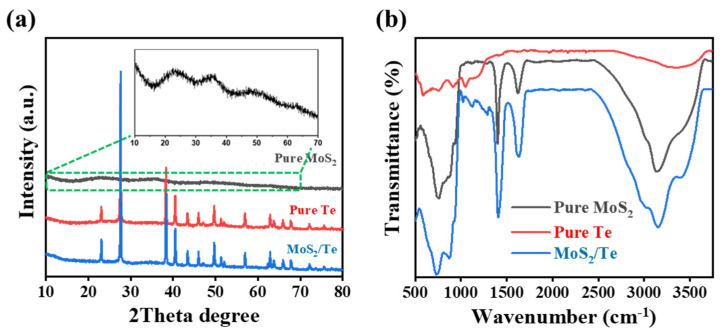
XRD and FTIR analyses of samples: (**a**) XRD spectra and (**b**) FTIR spectra.

XRD spectra were taken to analyze the structural crystallinity and phase purity of the as-synthesized nanocomposites, as shown in Figure 3a. MoS_2_ nanosheets showed broad diffraction peaks at (22.88 and 35.91)°, which respectively correspond to the (002) and (100) crystal planes (JCPDS code: 024-0513). The pure Te NRs showed distinct peaks at (27.57, 38.3, and 40.47)°, respectively corresponding to the (101), (012), and (110) peaks of the hexagonal crystal structure of Tellurium (JCPDS code: 01-086-2269). The as-synthesized final composite MoS_2_/Te showed the presence of characteristic peaks of both components, thus demonstrating the successful formation of the desired MoS_2_/Te hetero-structured nanocomposite. The nanocomposite was fabricated using a simple one-pot hydrothermal process at 195 °C. This fabrication temperature mostly leads to the production of 2H phase of MoS_2_ nanosheets. If a nanocomposite fabricated in this way is calcined at a higher temperature of around 1000 °C, then the 1T phase of MoS_2_ exists. As indicated in the XRD and XPS data, our nanocomposite mostly possesses 2H MoS_2_ phase with low or no 1T phase.

Similarly, Figure 3b presents the FTIR spectra of the synthesized nanocomposite. The absorption peaks at (1409, 1120, 875, and 751) cm^−1^ are attributed to MoS2 [36,37]. A broad peak at around 3367.8 cm^−1^ indicates the presence of the hydroxyl group of water molecules, while the peak corresponding to 1630 cm^−1^ is the indicative peak for the presence of water molecules in the nanocomposite. Lastly, the peak appearing at 586 cm^−1^ indicates the presence of Te–O stretching bond vibration [38]. These results further support the successful synthesis of the MoS2/Te hetero-structured nanocompo-site.

**Figure 4:**
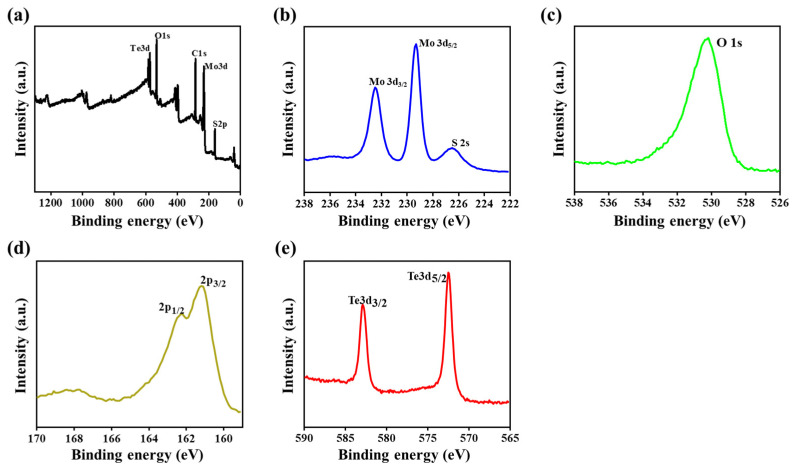
XPS spectra: (**a**) Survey spectrum, (**b**) Mo 3d, (**c**) O 1s, (**d**) S 2p, and (**e**) Te 3d.

Figure 4 shows the surface electrochemical composition and bonding nature of the component elements in the composite that were examined through X-ray photoelectron spectroscopy (XPS). Figure 4a shows the low-resolution wide-scan survey spectrum, which reveals the presence of peaks at the binding energies of (161.32, 231.91, 530.29, and 572.43) eV, corresponding to the elements with their respective orbitals, such as S 2p orbital, Mo 3d orbital, O 1s orbital, and Te 3d orbital, respectively, of the hetero-composite. The high-resolution XPS spectra of the individual elements and their orbital positions were recorded, as well. Figure 4b shows the Mo 3d orbital peaks at (232.48 and 229.31) eV, respectively corresponding to the Mo 3d_3/2_ orbital and Mo 3d_5/2_ orbital doublet peaks that are due to the +4 oxidation state of molybdenum (Mo^4+^) in MoS_2_, and the peak at 226.49 eV that is assigned to the S 2s orbital of MoS_2_ [22,39]. A very small amount of molybdenum exists in the (VI) oxide form, which indicates the presence of the p-orbital of the oxygen element, with the peak appearing at 530.19 eV assigned to the 1s orbital of oxygen (Figure 4c). Similarly, the doublet peaks of the p-orbital of Sulphur element that are observed at (162.23 and 161.14) eV are respectively correlated with the 2p_1/2_ and 2p_3/2_ orbitals of Sulphur element, as shown in Figure 4d [40]. Similarly, the 1s orbital of oxygen atom shows the peak position at 530.19 eV. The electrons present in the d-orbital of tellurium show peaks appearing at (582.91 and 572.46) eV, which are respectively attributed to the Te 3d_3/2_ and Te 3d_5/2_ orbitals, as shown in Figure 4e.

**Figure 5:**
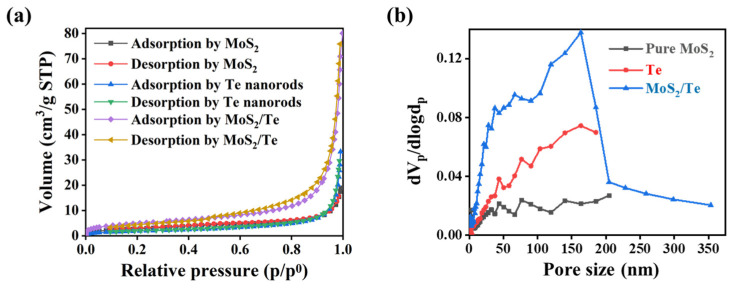
Surface areas and pore sizes of samples: (**a**) N_2_ adsorption–desorption isotherms and (**b**) pore size distribution curves for different samples.

The nitrogen adsorption–desorption technique at 77 K was used to investigate the specific surface areas and porous structures of the samples, as shown in Figure 5. According to the IUPAC technical report evaluating the surface area and pore size distribution, pure MoS_2_ and pure Te samples showed the type II physisorption isotherm, as presented in Figure 5a. This type of isotherm results from unrestricted monolayer–multilayer adsorption up to a high p/p^0^ value, which indicates a significant amount of overlap of the monolayer, which is attributed to the onset of multilayer adsorption. Typically, the thickness of the adsorbed multilayer increases without limit at p/p^0^ = 1. The final nanocomposite MoS_2_/Te shows the type IVa physisorption isotherm and the H3 hysteresis loop, and it indicates the formation of meso- and micro-porous structures. This isotherm is observed when the pore width exceeds a certain critical width, which is dependent on the adsorption system and temperature [41]. Similarly, Figure 5b shows the Barrett-Joyner-Halenda (BJH) curves that were obtained to show the pore size distribution of the samples. These show that the majority of pores lie in the range from 0–50 nm, indicating the presence of micro- and meso-pores in the samples. In addition, the final nanocomposite shows unique meso- and micro-porous structures with a high specific surface area, which could improve the easy electron transfer during electrochemical performance when used as an electrode material. This might be an extra benefit of the electrode material during electrochemical applications [42]. Table 1 lists the BET specific surface area, average pore size, and pore volumes of the samples. The nanosheets and nanorods with a homogenous smooth surface provide low specific surface areas, whereas loading the nanorods or nanoparticles on the surface of the nanosheets imparts a heterogenous rough surface structure which helps increase the specific surface area of the composite system. Adjoining the nanorods on the surface of the nanosheets during the formation of the composite significantly affects the surface roughness, ultimately leading to an improved specific surface area. These show that the incorporation of Te onto MoS_2_ results in the enhancement of specific surface area, which might be due to the typical roughness generated by the heterostructures of the nanocomposite.

### 3.2. Electrochemical Performances

The electrochemical activities of the as-prepared nanocomposites were analyzed through cyclic voltammetry (CV), galvanostatic charge discharge (GCD), and electrochemical impedance spectroscopy (EIS) on the nanocomposite-coated Ni-foam as the working electrode, Ag/AgCl as the reference electrode, and Pt-wire as the counter electrode, with 2 M KOH as the electrolyte. The CV profile was investigated with different scan rates ranging from (10–100) mV/s and the potential window from (0.0–0.6) V vs. Ag/AgCl reference electrode. 

**Figure 6:**
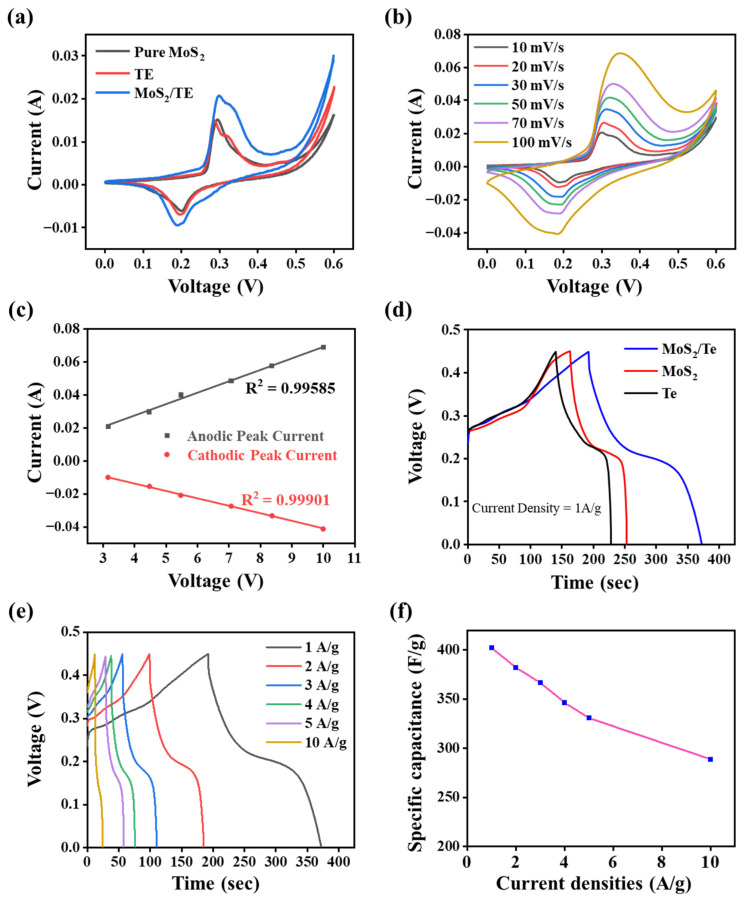
Electrochemical performances: (**a**) CV profiles for different nanoparticles and composites (scan rate: 10 mV/s, potential: (0–0.6) V, and ref. electrode: Ag/AgCl), (**b**) CV profiles for MoS_2_/Te at different scan rates, (**c**) anodic and cathodic peak current vs. Sq. root of scan rates, (**d**) GCD profiles of different nanoparticles and composites, (**e**) GCD profiles for MoS_2_/Te at different current densities, and (**f**) specific capacitance of MoS_2_/Te at different current densities. The data are presented as the mean ± SD (*p* < 0.05).

Figure 6a demonstrates the redox-type CV curves of the pure MoS_2_, Te NRs, and MoS_2_/Te nanocomposite at the scan rate of 10 mV/s obtained under similar conditions, which indicate the Faradaic charge storage mechanism. These curves clearly illustrate the larger integrated area with higher current range of the CV curve for the MoS_2_/Te nanocomposite, compared to its pure constituent electrode material, which is due to the collective role of both of the component elements present in the nanocomposite. CV curves of Te and MoS_2_/Te show the presence of double peaks. There are a number of different factors that can substantially alter the diffusion of electrons during the forward and backward potential sweep during the experiment, such as the heterogenous surface structure of the nano or microparticles, band width, and geometry of the electrode materials [43]. Furthermore, the CV performance of MoS_2_/Te was recorded at different scan rates ranging from (10 to 100) mV/s within the potential range from (0.0 to 0.6) V vs. the Ag/AgCl reference electrode. This produced the typical CV curves shown in Figure 6b. Here, all the CV curves show a pair of reversible redox peaks, which reveal significant differences compared to the electrical double-layer capacitors (EDLC, with ideal rectangular curves). This supports the pseudo-capacitive properties of the as-synthesized MoS_2_/Te hetero-composite electrode material [44]. The scan rate dependency of the redox current peaks from the CV profile was observed, as well. This shows that the redox current peaks were linearly proportional to the square root of the scan rates, as shown in Figure 6c.

Furthermore, the as-fabricated electrode materials were further investigated using GCD measurements taken at various current densities within the potential range from (0.00 to 0.45) V vs. Ag/AgCl reference electrode in 2 M KOH electrolyte. Compared to the potential window of the material during CV, the GCD profile shows a slightly narrower range of potential window, which is due to the evolution of gas at low current density. In addition, the GCD curves distinctly demonstrate deviations from the linear charge-discharge curves of the electrochemical double layer capacitor (EDLC), which are also supported by the pseudo-capacitive nature of the material, which is similar to that of the CV profiles, as discussed above. This property is caused by the redox reactions through the adsorption and desorption phenomena of hydroxyl ions within alkaline electrolyte to the electrode interface [45]. With higher scan rates, the oxidation and reduction peaks respectively move to negative and positive potentials, during which the rate of the ion diffusion must be limited to peruse the electronic neutralization of redox reactions. At a lower scan rate, the OH^−^ ions have a sufficient time period to intercalate with the inner and outer surfaces of the electrode. By contrast, at a higher scan rate, the OH^−^ ions do not have sufficient time for such intercalations within the inner and outer surfaces of the electrode. 

Figure 6d shows the charge-discharge profile of three different materials at a current density of 1 A g^−1^. The figure shows that the GCD profile of MoS_2_/Te has a higher discharge time than the other electrode materials, which is consistent with the CV profile of the same electrode material. The calculation of specific capacitance using the discharge time period at the current density of 1 A g^−1^ for the different electrode materials shows the specific capacitances of (195.55, 202, and 402.53) F g^−1^ for MoS_2_, Te NRs, and the MoS_2_/Te final nanocomposite, respectively. Furthermore, the specific capacitance of the MoS_2_/Te was calculated at different current densities ranging from (1 to 10) A g^−1^, as shown in Figure 6e. From the GCD profile, the specific capacitance for MoS_2_/Te was calculated for the different current densities of (1, 2, 3, 4, 5, and 10) A g^−1^, which were obtained as (402.53, 382.41, 367.76, 346.56, 331.00, and 289.87) F g^−1^, respectively. Figure 6f presents the specific capacitance against different current densities for MoS_2_/Te. The figure also shows that, even at the higher current density of 10 A g^−1^, the MoS_2_/Te composite retains a good rate performance of 72.01%. With the increasing current density, the specific capacitance gradually decreases, which might be due to the variations in the ion diffusion rates. The electrolyte ions also get enough diffusion time to the inner pores of the electrode surfaces for the optimum utilization of the active surface site of the electrode materials [46]. Table 2 presents some recent related works for comparative study. 

**Figure 7:**
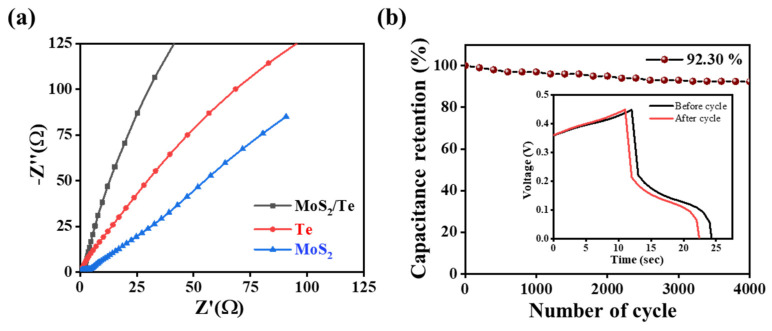
Electrochemical impedance spectroscopy studies of samples: (**a**) Nyquist plots of the different electrode materials recorded at (0.01 Hz to 100 kHz) and (**b**) cycling stability up to 4000 cycles.

The observed higher capacitive performance of the as-fabricated electrode material was further confirmed with the aid of EIS data. Figure 7a shows the Nyquist plots for all the three electrode materials recorded with 5 mV amplitude over the frequency range from (0.01 Hz to 100 kHz) associated with open circuit potential (OCP), a clear variation among the different electrodes can easily be visualized. The three plots show the arc patterns of the Nyquist plot in the high-frequency region, while they show a linear pattern in the low-frequency region. The semicircular arc type of the Nyquist plot is correlated with the Faradaic reactions, and its diameter is related to the intrinsic resistance (R_S_), which is further related to the combined resistance of the electrolyte and electrode materials. Similarly, the charge transfer resistance (R_CT_) arises from the electronic and ionic resistance at the electrode–electrolyte interface, while the Warburg diffusion resistance (R_W_) is generated from the resistance of ionic diffusion in the electrolyte and depends on the frequency [54,55]. Here, the intrinsic resistance (R_S_) is independent of the current density, and it typically remains constant for the given electrolyte and electrode material. 

A quantitative calculation of R_CT_ is used to capture the electrochemical activities of the electrode material [56]. The EIS plot of MoS_2_/Te shows a significant result with a semicircle at the high-frequency region, as well as an almost vertical trend at the low-frequency region, which can be related to the good extent of the capacitive activities. The smaller semicircular diameter and higher slope of the vertical line of MoS_2_/Te correspond to the small charge transfer resistance leading to the higher conductivity and low electrical impedance, which ultimately show better capacitive performance compared to the pure Te NRs and pure MoS_2_. Based on the Nyquist plot of the different electrode materials, the charge transfer resistances (R_CT_) for MoS_2_, Te NRs, and MoS_2_/Te composites were found to be 2.18, 1.87, and 1.01 Ω, respectively. Figure S2 illustrates the cyclic stability of the as-fabricated MoS_2_/Te composite electrode tested continuously for 4000 cycles of the charge-discharge profile at a current density of 10 A g^−1^. This stability performance retains the capacitive activity up to 92.30%, even after 4000 charge-discharge cycles, thus confirming the sufficient stability and reversibility of the MoS_2_/Te electrode composite (Figure 7b). The superior capacitive performance of this electrode material might be attributable to the good synergistic effect of the components of heterostructures, low interfacial charge transfer resistance between electrolyte and electrode surface, and fast and reversible electrode–electrolyte interfaces.

## 4. Conclusions

This work presents a facile one-pot hydrothermal method for the synthesis of MoS_2_/Te hetero-structured nanocomposite. The in situ hydrothermal reaction of the respective precursor elements successfully produced hetero composite of MoS_2_ conjugated with Te nanorods, the synergistic effect of which allows it to be utilized as a significant electrode material for an effective supercapacitor device. The various electrochemical profiles recorded for the as-fabricated MoS_2_/Te electrode material show the high specific capacitance of 402.53 F g^−1^ at a current density of 1 A g^−1^ as well as a good rate performance of 72.01%. This electrode material delivered excellent electrochemical stability of 92.30% of the initial specific capacitance, even after the continuous recording of the charge-discharge profiles up to 4000 cycles. This indicates that the prepared electrode material is a promising electrode for use as a material in supercapacitors. The simple hydrothermal technique of its preparation could be scaled up for large-scale preparation of the electrode material for its significant application to energy storage devices. Moreover, the application of this electrode material could also be studied and extended for use in data storage devices, lithium–ion batteries, and catalysts.

## Figures and Tables

**Table 1:** Comparative study of the BET parameters of the samples.

Samples	BET Specific Surface Area (m^2^ g^−1^)	Total Pore Volume (V_T_, cm^3^ g^−1^)	Mean Pore Diameter (nm)
MoS_2_	6.992	0.049	28.538
Te	10.513	0.027	10.426
MoS_2_/Te	18.212	0.110	24.286

**Table 2:** Comparison of recent composite electrodes based on electrolyte, test conditions, specific capacitance, and capacitance retention.

Composite Electrode	Electrolyte	Test Condition	Specific Capacitance	Capacitance Retention	Ref.
MoS_2_/rGO	1 M H_2_SO_4_	0.5 A g^−1^	365 F g^−1^	83% after 5000 cycles	[47]
MoS_2_/rGO	1 M Na_2_SO_4_	1 A g^−1^	198.6 F g^−1^	–	[48]
MoS_2_/CoS_2_/rGO	2 M KOH	0.5 mA cm^−2^	190 mF cm^−2^	88.6% after 5000 cycles	[49]
rGO-Co_3_O_4_	1 M KOH	200 mA g^−1^	278 F g^−1^	91.6% after 2000 cycles	[50]
Nb_2_O_5_@C/rGO	1 M NaPF_6_	0.025 A g^−1^	285 mA h g^−1^	92% after 1000 cycles	[51]
MoS_2_	1 M Na_2_SO_4_	1 A g^−1^	138 F g^−1^	86% after 5000 cycles	[19]
p-C_3_N_4_-Ti_2_CTx	6 M KOH	1 A g^−1^	327 F g^−1^	96.2% after 5000 cycles	[52]
V_2_CTxMXene	Seawater	0.2 A g^−1^	181.1 F g^−1^	89.1% after 5000 cycles	[49]
NiSe_2_	1 M KOH	1 mA cm^−2^	75 F g^−1^	94% after 5000 cycles	[53]
MoS_2_/Te	2 M KOH	1 A g^−1^	402 F g^−1^	92.30% after 4000 cycles	This work

## Data Availability

The data presented in this study are available on request from the corresponding author.

## Supplementary Materials

The following are available online at https://www.mdpi.com/article/10.3390/nano11092346/s1. Figure S1: energy dispersive spectroscopy (EDS) analysis; Figure S2: EIS profile of MoS_2_/Te before and after the 4000 cycles.
