# Supplementary material for: Synthesis of Molybdenum Sulfide/Tellurium Hetero-Composite by a Simple One-Pot Hydrothermal Technique for High-Performance Supercapacitor Electrode Material
Source: Nanomaterials (Basel). 2021 Sep 9;11(9):2346. doi: 10.3390/nano11092346 (PMC8468014; doi:10.3390/nano11092346)
Supplement: Supplementary file 1 [file nanomaterials-11-02346-s001.zip › nanomaterials-1357357-supplementary.pdf]

Supplementary materials

Synthesis of Molybdenum Sulfide/Tellurium Hetero-Composite by a Simple One-Pot Hydrothermal Technique for High-Performance Supercapacitor Electrode Material

Hem Prakash Karki [1,3,†], Hyojae Kim [1,†], Jinmu Jung [1,2,*] and Jonghyun Oh [1,2,*]

1. Department of Mechanical Design Engineering, College of Engineering, Jeonbuk National University, Jeonju 54896, South Korea; hyojaekim@jbnu.ac.kr
2. Department of Nano-bio Mechanical System Engineering, College of Engineering, Jeonbuk National University, Jeonju 54896, South Korea
3. Department of Environmental Engineering, Korea University, 2511 Sejong-ro, Sejong City 30019, South Korea; hpkarki666@gmail.com (Current address)

* Correspondence: jmjung@jbnu.ac.kr (J.J.), jonghyuno@jbnu.ac.kr (J.O.)

† These authors contributed equally to this work.

Materials and methods

Fabrication of working electrode

Ni-foam was cut into squares of 1 × 1 cm^2 in dimension, and it was first treated with 3 M HCl solution under ultrasonication for 30 min to remove any oxide film present on its surface. Again, it was treated with acetone, and ultrasonicated for 1 h in the water bath. Then the treated Ni-foam was washed with DI-water several times, followed by absolute alcohol to ensure the surface of Ni-foam was cleaned. Finally, the clean piece of Ni-foam was dried in vacuum oven at 60 °C for 6 h. The working electrode was fabricated with 4 mg of material mixture, where the as-prepared nanocomposite was taken as exactly 3.2 mg, 0.4 mg of activated carbon, and 0.4 mg of polyvinylidine fluoride (PVDF) in the mass ratios 80:10:10. The mixture content was poured in mortar, where N-methyl-2-pyrrolidone (NMP) was added and mixed thoroughly to make the homogenous slurry. All of this nanocomposite slurry was slowly drop cast over the surface of the cleaned Ni-foam substrate, and finally kept in the oven at 70 °C for 12 h.

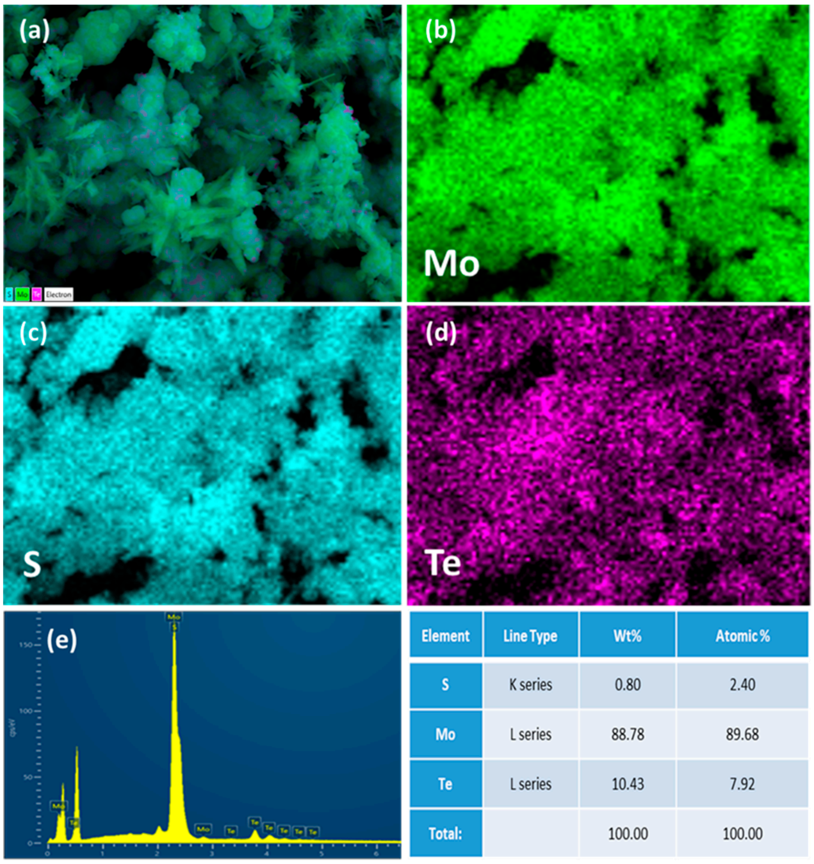

Element	Line Type	Wt%	Atomic %
S	K series	0.80	2.40
Mo	L series	88.78	89.68
Te	L series	10.43	7.92
Total:		100.00	100.00

Figure S1. Energy Dispersive Spectroscopy (EDS) analysis: (a) MoS_2/Te hetero-composite, (b) Mo, (c) S, (d) Te, and (e) elemental spectrum and table for the elemental composition of the MoS_2/Te nanocomposite.

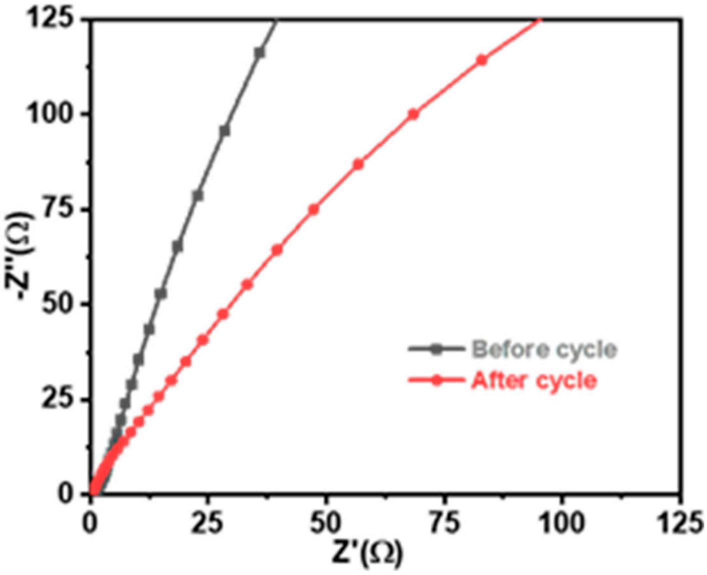

Figure S2. EIS profile of MoS_2/Te before and after the 4,000 cycles.
